# Scaffolded Antigens in Yeast Cell Particle Vaccines Provide Protection against Systemic Polyoma Virus Infection

**DOI:** 10.1155/2016/2743292

**Published:** 2016-04-26

**Authors:** Donald J. Tipper, Eva Szomolanyi-Tsuda

**Affiliations:** ^1^Department of Microbiology and Physiological Systems, University of Massachusetts Medical School, Worcester, MA 01655, USA; ^2^Department of Pathology, University of Massachusetts Medical School, Worcester, MA 01655, USA

## Abstract

*Background*. U65, a self-aggregating peptide scaffold, traps fused protein antigens in yeast cells. Conversion to Yeast Cell Particle (YCP) vaccines by partial removal of surface mannoproteins exposes *β*-glucan, mediating efficient uptake by antigen-presenting cells (APCs). YCP vaccines are inexpensive, capable of rapid large-scale production and have potential for both parenteral and oral use.* Results*. YCP processing by alkaline hydrolysis exposes up to 20% of the glucan but converts scaffolded antigen and internal yeast proteins into a common aggregate, preventing selective yeast protein removal. For U65-green fluorescent protein (GFP) or U65-Apolipoprotein A1 (ApoA1) subcutaneous vaccines, maximal IgG responses in mice required 10% glucan exposure. IgG responses to yeast proteins were 5-fold lower. Proteolytic mannoprotein removal produced YCPs with only 6% glucan exposure, insufficiently porous for selective removal of even native yeast proteins. Vaccine efficacy was reduced 10-fold. Current YCP formulations, therefore, are not suitable for human use but have considerable potential for use in feed animal vaccines. Significantly, a YCP vaccine expressing a GFP fusion to VP1, the murine polyoma virus major capsid protein, after either oral or subcutaneous administration, protected mice against an intraperitoneal polyoma virus challenge, reducing viral DNA levels in spleen and liver by >98%.

## 1. Introduction

Glucan particles (GPs), prepared from the walls of* Saccharomyces cerevisiae* cells, are composed primarily of *β*-1,3-D-glucans (*β*G) [1, 2]. These are recognized as a fungal cell wall pathogen-associated molecular pattern (PAMP) and GPs serve as an effective adjuvant for IgG antibody production when admixed with free antigens [3]. Host phagocytic receptors, including dectin-1 [4] and complement receptor 3 [5], recognize fungal *β*G and this interaction leads to enhanced cytokine responses by innate immune cells, contributing to GP adjuvant function [2, 6, 7]. The particles have far broader efficacy when the antigen is encapsulated inside GPs, ensuring codelivery of both glucan adjuvant and antigen to the same endosomal compartment in the same antigen-presenting cell (APC) [3]. Robust antibody and both Th1- and Th17-biased CD4 T cell responses result [3].

Recombinant yeast cells expressing protein antigens should have similar advantages for codelivery of antigen and adjuvant, and several reports describe their use as oral vaccines in feed animals [8].* Saccharomyces cerevisiae* (baker's yeast) was chosen for antigen expression and YCP vaccine production since it is generally regarded as safe for human use (GRAS). In intact yeast cells, however, the highly porous inner cell wall *β*-glucan matrix [3] is masked by a dense surface mannoprotein layer [9]. Interactions with mannan receptors provide only inefficient phagocytosis. If mannoproteins can be effectively removed, eliminating most of these immunodominant yeast antigens [10] while exposing glucan and retaining antigen, the resultant Yeast Cell Particles (YCPs) should constitute an inexpensive, effective vaccine, capable of rapid production in large quantities and easily stored as dry powder without refrigeration. YCP vaccines could potentially be used either parenterally or orally, in enteric-coated pill form or as a feed additive.

**Figure 1:**
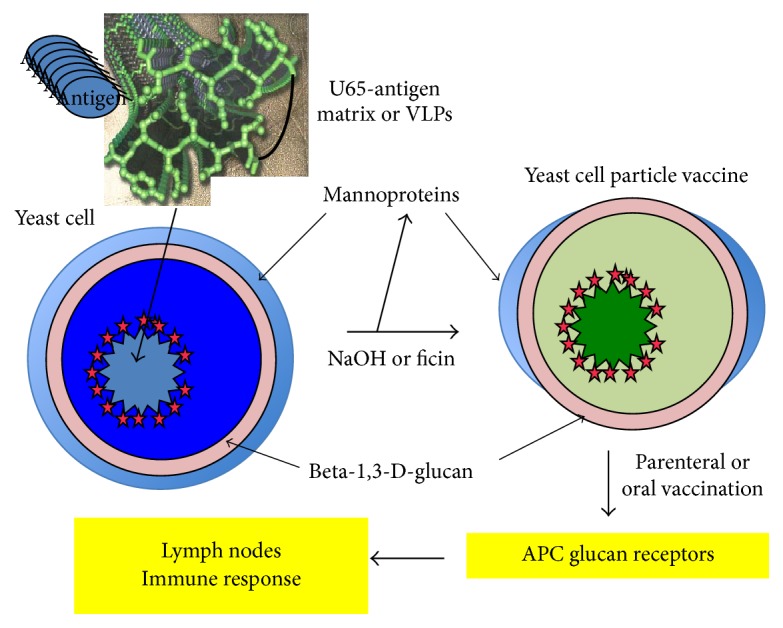
YCP vaccine processing and function.

Achievement of this aim poses several requirements. First, while* S. cerevisiae* has frequently been used for expression of foreign proteins, levels are generally low and routine expression levels of at least 5–10% of total protein would be desirable.* Pichia pastoris* can be used for much more efficient protein expression and has been used to produce vaccines tested in mice [11] and chickens [12] but is not GRAS. Second, a simple and reproducible extraction method needs to be developed to process cells into YCP vaccines, exposing a sufficient fraction of cell wall *β*G to ensure efficient phagocytosis and removing most yeast mannoprotein antigens [10]. Third, this process should allow retention of the desired antigen and, preferably, should selectively remove internal yeast proteins. While the outer mannoprotein cell wall layer is relatively impermeable, the glucan matrix in GPs is porous to even large proteins [3]. To ensure antigen retention, therefore, it seemed likely that it would be necessary to trap the antigen either linked to a self-assembling polymeric peptide aggregate or as self-assembling virus-like particles (VLPs). Finally, vaccine processing (Figure 1) should sterilize the YCP product. As described below, all objectives have been achieved except for selective antigen enrichment by removal of internal yeast host proteins.

While VLPs have been used to present antigenic peptides in yeast [11], VLP scaffolds may present competing epitopes. We chose to express antigens as fusions to U65, the weakly antigenic 65-residue N-terminal fragment of the yeast protein Ure2p. Polymerization of U65 is self-propagating and overexpression of U65 is sufficient to induce polymerization as cytoplasmic fibrils [13]. It was anticipated that U65 fusions to antigens that do not spontaneously aggregate, such as green fluorescent protein (GFP), would produce stable cytoplasmic aggregates with enhanced antigenicity and improved retention during YCP vaccine preparation. The lowest percentile rank MHCII binding predicted for U65 in C57BL/6 mice [14, 15] was 12.3, so U65 epitopes should not compete with responses to coupled antigens with stronger epitopes. The U76L scaffold contains 11 additional Ure2p residues, potentially enhancing scaffold stability, and “L,” a 9-residue flexible linker allowing independent folding of U76 and any fused antigen. Predicted MHCII binding is unchanged. Both U65 and U76L were used as scaffolds for expression of model antigens GFP (26.9 kDa) and mature human Apolipoprotein A1 (ApoA1, 28.3 kDa). In this paper, we demonstrate that YCPs made from both U65- and U76L-GFP and ApoA1 fusions were highly effective in eliciting IgG responses in mice and determine the minimal % glucan exposure sufficient for optimal immune responses.

As a model for the use of YCP vaccines for the prevention or treatment of infectious disease, we vaccinated mice with YCPs expressing a GFP fusion to VP1, the major capsid protein of mouse polyomavirus (MPyV), which, like the hepatitis B surface antigen (HBsAg), forms VLPs when expressed in yeast [16–18], even when C-terminally fused to large antibody fragments [19]. We demonstrate that the VP1-GFP fusion assembles into VLP-like structures in the yeast cytoplasm and that both subcutaneous and oral administration of a YCP-VP1-GFP vaccine resulted in effective protection against systemic infection with MPyV.

## 2. Materials and Methods

### 2.1. Animals

This study was performed in strict accordance with the* NIH Guide for the Care and Use of Laboratory Animals*. The protocols were approved by the University of Massachusetts Medical School Institutional Animal Care and Use Committee (docket A-1778). Mice were observed daily and euthanized by CO_2_ overdose, followed by cervical dislocation.

### 2.2. Yeast Strains and Media

*Saccharomyces cerevisiae* strain CRY1 has the genotype MAT*α ura3-1 trp1-1 leu2-3,112 his3-11 can1-100 ade2 GAL SUC* [20]. Strain PAP1502 has the genotype MAT*α ura3-52 trp1::GAL10-GAL4 lys2-801 leu2Δ1 his3Δ200 pep4::HIS3 prb1Δ1.6R can1 GAL* and was kindly provided by Dr. Pedersen et al. [21]. Yeast extract-glycerol-peptone (YGP), uracil drop-out (Ura D/O), uracil + leucine drop-out (Ura + Leu D/O), and uracil + tryptophan drop-out (Ura + Trp D/O) media were prepared as described in Cold Spring Harbor protocols (Cold Spring Harbor Press).

### 2.3. Antisera and Antiglucan FACS Assay for Glucan Exposure

Anti-*β*-1,3-glucan antibody murine monoclonal IgG kappa was purchased from Biosupplies, Australia. For analysis of glucan exposure, yeast cells, YCPs, or glucan particles (4 × 10^7^) were washed in phosphate-buffered saline (PBS), suspended in 500 *μ*L PBS + 1% bovine serum albumin (BSA) for 30 min at 23°C, and then suspended in 100 *μ*L anti-*β*-1,3-glucan mouse IgG diluted 1 to 100 in PBS + 1% BSA. After 60 min at 23°C, cells were washed three times in PBS + 1% BSA and suspended in R-phycoerythrin-conjugated polyclonal goat anti-mouse IgG (Sigma-Aldrich) diluted 1 to 50 in PBS + 1% BSA. After 60 min, cells were washed three times in PBS + 1% BSA and analyzed by fluorescence-activated cell sorting (FACS).

### 2.4. Concanavalin A Assays for Exposed Mannan

Cells and YCPs were incubated with various dilutions of Concanavalin A-FITC (fluorescein isothiocyanate) conjugate (Sigma-Aldrich) or Concanavalin Alexa Fluor 488 (Life Technologies) in PBS. After 5 min at 23°C, cells were washed three times in PBS and assayed by fluorescent microscopy (FITC) or by measurement of the 488/519-excitation/emission fluorescence signal. GPs were used as negative controls and YCPs were compared to untreated cells with 100% normal mannan exposure.

### 2.5. 3T3-D1 Cell Phagocytosis

NIH 3T3-D1 cells [4] were plated at 2.5 × 10^4^ cells/well in 24-well plates in 0.5 mL DMEM (Invitrogen) + 10% fetal calf serum (Gibco) + 1% penicillin-streptomycin (Gibco) and incubated overnight at 37°C, 5% CO_2_, producing 5 × 10^4^ cells/well. Wells were washed with PBS (Gibco) and 500 *μ*L DMEM-minus serum was added. Cells or YCPs were washed in PBS, diluted to 5 × 10^6^/mL, and 50 *μ*L was added to wells to achieve a ratio of 5 particles/cell. After incubation for 1.5 hours, cells were washed twice with PBS, fixed in 1% formalin, and stained with Congo red (Sigma-Aldrich), which binds to yeast glucan. Cells were then scored microscopically at 200x magnification for evidence of yeast particle phagocytosis by detection of GFP and coincident staining with Congo red. Because untreated yeast cells adhere to 3T3 cell surfaces, backgrounds were high (25%) and only responses >50% were scored as positive.

### 2.6. Plasmid Constructs

*pB4-VP1*. The HBsAg open-reading frame (ORF) and the preceding* pGAL/pGDH* promoter were cloned by PCR from* S. carlsbergensis* strain ATCC 20705 total DNA using the primers 5′ GGG** AAG CTT** CTC TTT GGA ACT TTC AG and 5′ GGG** AGA TCT** CAA TAA GAG CGA CCT CAT GC. The product was cloned as a 1450 bp HindIII to BglII fragment in p-M28 LEU2 [22], producing pB4-HBs. The VP1 capsid protein gene of MPyV, nominally strain A2, was cloned by PCR using as template a tissue culture supernatant from infected cells and the primers 5′  C ACC  AAG AAC TTA GTT TCG AAT AAA CAC ACA TAA ACA AAC AA**C TCG AG**G AAG  ATG GCC CCC AAA AGA AAA AGC  and 5′ TTG ATC TAT CGA TTT CAA TTC AAT TCA ATT TAT TTC CCG GAT CGA** AGA TCT** CAT TAA TTT CCA GGA AAT ACA GTC TTT G. The flanking sequences are homologous to the ends of the pGDH and tPGK segments in pB4-HBs. pB4-HBs was cut with XbaI (at codon 31 in HBsAg) and BglII and the purified vector fragment and PCR product were cotransformed into* leu2* yeast strain CRY1. Sequencing confirmed that Leu+ isolates carried pB4-VP1 in which the VPI open-reading frame replaces HBsAg between the* pGAL/pGDH* promoter and the* tPGK* terminator. Sequence analysis showed that the cloned VP1 open-reading frame is precisely that from strain BG (GenBank AF442959).

**Figure 2:**
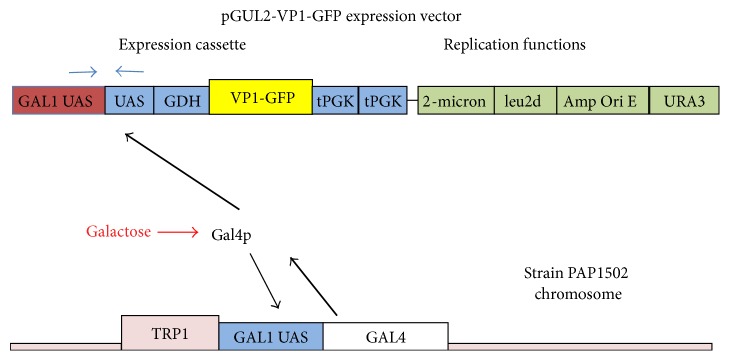
The pGUL2-VP1-GFP expression vector showing the expression cassette and its induction by galactose-induced expression of Gal4p, augmented by overexpression from the GAL1 UAS-driven GAL4 gene inserted at TRP1 in the strain PAP1502 chromosome.

**Figure 3:**
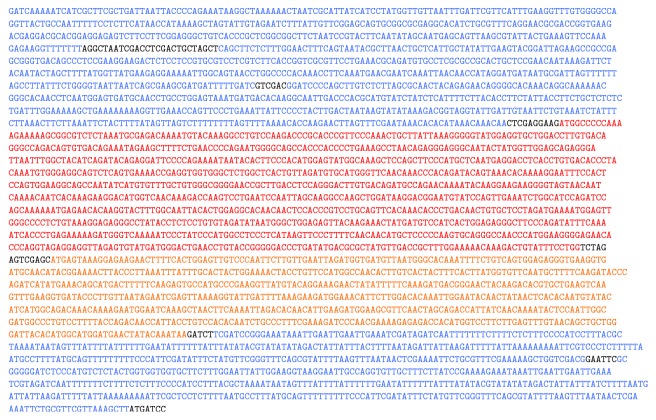
Sequence of the pGUL2-VP1-GFP expression cassette showing the duplicated upstream GAL1/10 UAS fragments (blue), the first (357 bp) in GAL1-GAL10 orientation and the second (353 bp) inverted. These are followed by the pGDH fragment (388 bp, also blue) completing the hybrid promoter. The reading frame for strain BG VP1 (1152 bp, red) is fused to GFPbex (915 bp, orange) and the two tPGK terminator segments (314 and 381 bp, blue) complete the cassette, as shown schematically in Figure 2.

*pB4-GFP, pGUL2-GFP, pGUL2-VP1, and pGUL2-VP1-GFP*. In this paper, GFP refers to GFPbex1, a form of GFP with 25-fold enhanced fluorescence [23]. GFPbex1 was cloned by PCR from pDJ388 [23] using primers 5′ G CAG GGC TCG AGC ATG AGT AAA GGA GAA GAA CTT TTC ACT G and 5′ GGA TCG GAG ATC TTA TTT GTA TAG TTC ATC CAT GCC ATG and cloned into pB4-VP1, replacing VP1 and producing pB4-GFP. The GFP-tPGK fragment of pB4-GFP was inserted into the expression cassette of pEMBLyex4 (EMBL, Heidelberg). The* CYC1*-GFP fragment was then replaced with the* GAL1-GDH* promoter and GFP segments from pB4-GFP producing pGUL2-GFP. The GFP fragment in pGUL2-GFP was replaced with the VP1 segment of pB4-VP1 producing pGUL2-VP1. The* GDH* fragment of pGUL2-GFP was replaced with the* GDH*-VP1 segment of pB4-VP1 producing pGUL2-VP1-GFP. As illustrated in Figure 2, all pGUL2 constructs comprise two copies of the bidirectional* GAL1/10* Gal4p binding sites (in inverted orientations). These precede the* GDH* promoter fragment and the expressed reading frames and are followed by two tPGK terminators. The sequence of the expression cassette in pGUL2-VP1-GFP, including the BG strain VP1 gene, is shown in Figure 3.

**Figure 4:**
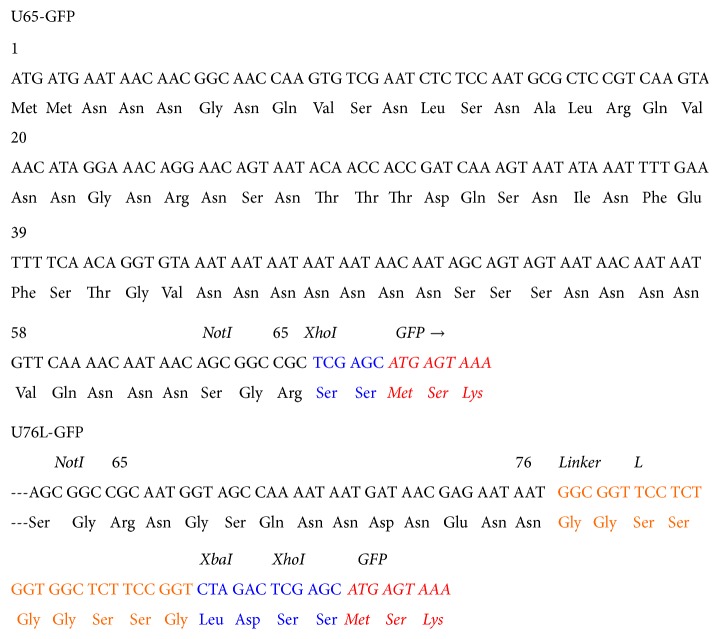
Sequences of the U65 and U76L scaffold fragments showing the XhoI sites (blue) for fusions to GFP (red) just upstream of the NotI site in U65 and following codon 76 and the 9-residue flexible linker (orange) in U76L.

*pGUL2-U65- and U76L-GFP*. The 65-codon N-terminal fragment of the yeast* URE2* protein Ure2p, including the native NotI site at its C-terminus, was cloned by PCR using pH324A [13] as template and the primers 5′ GGG TAG CCT CGA** GGA TCC GTC GAC** AGA  ATG ATG AAT AAC AAC GGC AAC  and 5′ GCT GCT** GAA TTC** TCG  A **GC GGC CGC** TGT TAT TGT TTT G. The product was cut with SalI and XhoI and cloned into pGUL2-GFP, cut with XhoI, and hydrolyzed with CIP, producing pGUL2-U65-GFP. Sequence analysis identified clones with 195 bp inserts corresponding to single U65 inserts linked in-frame to GFP (Figure 4). The 76-codon N-terminal fragment of Ure2p was cloned using pH324A [13] as template and the primers 5′ GG GTA GC**G TCG ACA CTA GT**C AGA  ATG ATG AAT AAC AAC GGC AAC  and 5′ CAG GAC C**CT CGA GTC TAG A**CC GGA AGA GCC ACC AGA GGA ACC GCC ATT  ATT CTC GTT ATC ATT ATT TTG. The product was cut with Sal1 and Xho1 enzymes and cloned into pGUL2-GFP, cut with Xho1, and hydrolyzed with CIP. Sequence analysis identified clones with 255 bp inserts corresponding to single U76L inserts linked in-frame to GFP. U76 and GFP are separated by the 9-residue L (GGSSGGSSG) linker segment (Figure 4).

*pGUL2-U65-ApoA1 and U76L-ApoA1*. The mature human Apolipoprotein A1 (ApoA1) gene was constructed by GeneArt Inc. with codons optimized for expression in yeast and cloned as an XhoI to BglII fragment into pGUL2-U65-GFP, cut with the same enzymes, replacing the GFP segment and producing pGUL2-U65-ApoA1. Insertion of the same ApoA1 fragment into pGUL2-U76L-GFP produced pGUL2-U76L-ApoA1. Insertion of an XbaI linker between the U65 and GFP components of pGUL2-U65-GFP and insertion of the ApoA1 gene as an XhoI to XbaI fragment produced pGUL2-U65-ApoA1-GFP. pGUL2-U76L-GFP was converted to pGUL2-U76L-ApoA1-GFP by the same procedure.

### 2.7. Protein Expression in pGUL2 Transformants of Strain PAP1502

pGUL2 vectors (Figures 2 and 3) carry both* URA3* and* leu2d* markers. While selection for URA3 function maintains 10–20 plasmid copies/cell, normal for YEp vectors with 2-micron replicative functions, selection for leu2d function requires at least 10-fold higher copy number, potentially allowing a similar increase in plasmid-encoded expression. All cultures were grown at 30°C. Transformants were selected on Ura D/O plates, cloned on these plates, and grown in Ura + Trp D/O medium with 2% sucrose as carbon source. The absence of Trp ensured maintenance of the* pGAL1*-driven* GAL4* gene integrated with* TRP1* at* trp1* (Figure 2) [21]. Under galactose induction, autoinduced Gal4p expression diverts most of the transcription/translation machinery to* GAL* genes. Cell division ceases, plasmid selection becomes irrelevant, and nonselective GP medium can be used to support galactose-induced expression. Expression of pGUL2-GFP protein fusions under* leu2d* selection then approaches 30% of total protein. Stationary phase cells were diluted 1 to 100 in prewarmed Leu + Trp D/O medium with 0.6% sucrose and 2% glycerol (500 mL) and grown in 3-liter fluted Fernbach flasks with high aeration for 18–20 hours, by which time they should be fully derepressed for* GAL* gene expression, reaching 6 × 10^7^ cells/mL. Cells were then rapidly harvested by centrifugation at 20°C, suspended in 250 mL of YGP medium at 30°C, and, after 60 minutes, induced by the addition of 27 mL of 20% galactose. Cell numbers remained constant. Cells were harvested after 18–22 hours.

### 2.8. SDS-PAGE and Determination of Expressed Protein Content by Stain or GFP Fluorescence

Cells and YCPs (1.2 × 10^7^) in 0.2 mL buffer A (50 mM Tris-HCl, pH 7.6, 150 mM NaCl, 10 mM NaN_3_, 10 mM KF, and 2.5 mM EDTA) were completely broken by vortexing with glass beads. Total proteins in these samples, or soluble proteins after removal of cell wall fragments by brief centrifugation at 3,000 ×g (3 to 12 *μ*L), were analyzed by 10% SDS-PAGE. Gels were stained with Coomassie blue and imaged using a Kodak Gel logic 200 illuminator. Exposures were adjusted to give a range of images representative of band densities. These were quantitated using Photoshop, giving the ratio of pixel density in individual bands to that in the entire gel lane. Pixel density at the same location in a lane on the same gel showing proteins from equal cell numbers expressing a protein with different mobility was subtracted. The difference is the % of total protein represented by the band of interest. Protein content of U65-GFP after purification from broken cells provided a standard for fluorescence analysis of GFP content, measured using a Molecular Devices microtiter plate reader (490 nm excitation, 520 nm emission). Fluorescence intensities of unbroken and broken cells were essentially identical, allowing determination of GFP fusion protein expression by measurement of culture fluorescence.

### 2.9. High pH YCP Vaccine Processing

Cells (6 × 10^10^) were washed by suspension in 500 mL sterile 0.9% saline and then three times in 60 mL saline. Cells, vigorously stirred at 10^9^/mL in 50 mL saline, were warmed to 45°C and then rapidly adjusted to pH 11.5–12 by addition of 4 M NaOH. The initial pH under standard conditions was 11.9. Additional 4 M NaOH was added, mostly in the first 3 min, to prevent the pH from falling more than 0.2 units below the initial pH to overcome the buffering resulting from exposure of intracellular components, principally phosphate esters (PK_a3_ 12.3). After 12 min, 1 mL of 1 M Tris-HCl was added, lowering the pH to about 9. Cells were then transferred to an ice/water bath, centrifuged at 4°C, washed four times in 50 mL cold PBS, and stored at −20°C in 5 mL aliquots (2.4 × 10^9^/mL).

### 2.10. Ficin YCP Processing

PAP1502 cells (4.8 × 10^9^/mL) were shaken at 37°C in 25 mL PBS + 10 mM EDTA, 4 mM azide, 1%  *β*-mercaptoethanol, and 8 mg/mL ficin (Sigma). After 30 min at 37°C, cells were washed three times at 23°C with saline, twice with 25 mL 0.02 M NaOH (pH of the suspension was 12), again with saline, then with PBS + 50 *μ*M TPCK (Sigma) to inactivate residual ficin, and finally three times with PBS. Ficin YCPs were stored at −20°C in PBS in 5 mL aliquots (2.4 × 10^9^/mL).

### 2.11. YCP U76L-ApoA1 Vaccination Protocol

A single group of four C57BL/6 female 6-week-old mice were vaccinated subcutaneously with 100 *μ*L (4 × 10^8^) YCPs containing about 120 *μ*g U76L-ApoA1 (90 *μ*g ApoA1) on days 0, 14, 28, and 42 and mice were bled on days 13, 27, 41, and 55. IgG responses to ApoA1 were determined by ELISA using wells coated with 0.1 *μ*g of ApoA1, a gift from Capricorn Products LLC. Wells coated with 0.1 *μ*g of purified U76L-GFP were used to test IgG responses to the U76L scaffold, and wells coated with 1 *μ*g of total proteins from PAP1502 cells expressing pGUL2-VP1 were used to test IgG responses to total yeast proteins.

### 2.12. U65-GFP Vaccination Protocol

25 C57BL/6 female 6-week-old mice were divided into five groups of five as described below. All mice were vaccinated subcutaneously on days 0, 14, 28, and 42 and bled on days 13, 27, 41, and 55. Sera were assayed for IgG responses by ELISA using wells coated with 0.1 *μ*g recombinant GST-GFP purified from* E. coli*. Mice other than Group E were vaccinated with 100 *μ*L YCPs (4 × 10^9^/mL), containing about 110 *μ*g U65-GFP (90 *μ*g GFP). Group A mice were vaccinated with U65-GFP YCPs extracted at 45°C, pH 11.9–11.7. Group B mice were vaccinated with YCPs extracted at 45°C at pH 11.6–11.4. Group C mice were vaccinated with YCPs extracted at 45°C at pH 11.5–11.3. Group D mice were vaccinated with YCPs processed using ficin. Group E mice were vaccinated at two separate sites, each with 100 *μ*L of the same U65-GFP YCP vaccine used for mice in Group A, but at 2x concentration (8 × 10^9^/mL), so that the total dose was 4x the Group A dose.

### 2.13. VP1-GFP YCP Oral and Subcutaneous Vaccination and Viral Challenge Protocols

Groups of five six-week-old C57BL/6 female mice were immunized with the YCP-VP1-GFP vaccine. Group A mice were immunized subcutaneously on days 1, 15, 29, 43, and 57 with 1.25 × 10^8^ YCPs in 50 *μ*L PBS containing about 800 *μ*g total protein, of which 96 *μ*g (12%) was the VPI-GFP fusion protein, 36 *μ*g was GFP, and 60 *μ*g was VP1. Oral dosage by gavage (Groups B and C) was administered to mice fasted for three hours, both before and after dosage, and in the presence of bicarbonate to suppress stomach acidity [24]. Group B mice were vaccinated by oral gavage with 5 × 10^8^ YCPs in 80 *μ*L PBS containing about 400 *μ*g VPI-GFP fusion protein (low dose, 250 *μ*g VP1) and Group C mice received 1.25 × 10^9^ YCPs in 250 *μ*L PBS containing 960 *μ*g VPI-GFP fusion protein (high dose, 600 *μ*g VP1). Group D mice were unvaccinated controls. Mice were orally vaccinated on three consecutive days (days 1, 2, and 3; 15, 16, and 17; etc.) at two-week intervals and so received either 12.5 or 30 times the subcutaneous dose. One mouse in each group was prebled on day 0 to provide preimmune sera. Mice were bled on days 14, 28, 42, 56, and 71 through tail veins. Stools were collected for IgA analysis on day 56. Mice were challenged intraperitoneally with 10^6^ plaque-forming units (pfu) of polyomavirus strain A2 in ~100 *μ*L volume on day 72 and were sacrificed for analyses of viral DNA content on day 77.

### 2.14. ELISA to Measure Antigen-Specific IgG Titers

MPyV VP1-specific ELISAs were conducted as previously described [25]. Briefly, recombinant VP1 protein produced in* E. coli* and highly purified (kind gift from Dr. Robert Garcea, University of Colorado, Boulder) was used to coat 96-well plates (at 0.1 *μ*g/mL in carbonate buffer, 50 ng/well). Purified recombinant GST-GFP fusion was used to coat wells used to assay responses to GFP. Bound antibody was detected using biotin-conjugated goat antibodies specific for mouse IgG and streptavidin-conjugated HRP (Vector Laboratories). ELISA plates were developed using BD OptEIA TMB Substrate Set (BD Pharmingen), and reactions were stopped with 2 N sulfuric acid. Optical densities were read at 450 nm using a Molecular Probes microplate reader and analyzed using Softmax software. ApoA1-specific, GFP-specific, and yeast protein-specific assays used plates coated with 0.1 *μ*g/well ApoA1 or GFP or 1 *μ*g/well total yeast proteins from strain PAP1502 cells, respectively. IgG titers are expressed as reciprocals of the highest serum dilutions that gave responses twofold above the negative controls plus standard deviation.

### 2.15. Quantitative PCR (qPCR) to Measure MPyV DNA Genome Copy Number

DNA was prepared from organ homogenates by digestion with proteinase K (Sigma) at 55°C overnight, followed by phenol extraction and RNase A treatment (10 u/mL, Promega) [25]. A 50 *μ*L PCR amplification mix contained 0.5 U of Taq polymerase (Promega), 0.66 U SYBR-Green (Molecular Probes), 0.1 mM each of forward and reverse primer (Invitrogen), 5 nM fluorescein (Bio-Rad), and 1 *μ*g of the DNA sample. Primers were as follows: *β*-actin forward CGA GGC CCA GAG CAA GAG AG; *β*-actin reverse CGG TTG GCC TTA GGG TTC AG; MPyV VP1 forward CCC CCG GTA CAG GTT CAG TCC CAT CAT; VP1 reverse GGC ACA ACA GCT CCA CCC GTC CTG CAG. Negative controls for PCR amplification of viral DNA included no DNA and DNA from uninfected mouse organs. Serial dilutions of DNA from uninfected mouse organs (1 mg–31 ng) were used to generate a *β*-actin standard curve. For MPyV standard curve, a plasmid containing the VP1 coding sequences in dilutions from 26108 to 20 copies was mixed with 1 *μ*g of DNA from uninfected mouse organs. Reactions were run in duplicate and MPyV copy number data were normalized to *β*-actin gene copies, reflecting the amount of mouse genomic DNA present, and the results were expressed as MPyV genome copies/*μ*g organ DNA. Statistical significance was determined by unpaired Student's *t*-test, assuming unequal variances. A *p* value of <0.05 was considered statistically significant.

## 3. Results

### 3.1. High Level Expression of U65-GFP and ApoA1 Fusions Generates Cytoplasmic Inclusions

**Figure 5:**
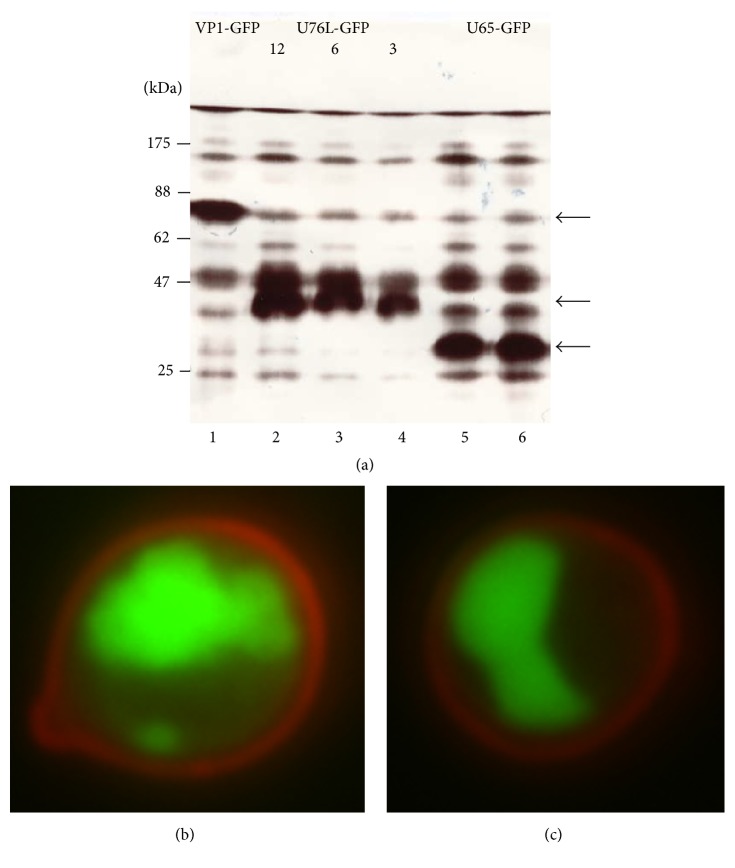
(a) SDS-PAGE of proteins from cells expressing the following: Lane 1, VP1-GFP (70 kDa, arrow); Lanes 2–4, U76L-GFP (36.2 kDa, arrow, loads of 12, 6, and 3 *μ*L, resp.); Lanes 5 and 6, U65-GFP (34.1 kDa, arrow, independent cultures). (b) Fluorescence microscopy of cells expressing U65-GFP. Cell wall glucan is stained by Congo red. (c) pH 11.5 YCPs.

pGUL2-antigen transformants of strain PAP1502 were used for all YCP vaccine production. Optimal expression of VP1-GFP (70 kDa), U65-GFP (34.1 kDa), and U76L-GFP (36.2 kD) was 30, 26, and 23% of total protein, respectively. These were easily visible on stained SDS gels of total proteins (Figure 5(a)). The background from overlapping yeast protein bands was subtracted in measuring fusion protein expression by stained band intensity. GFP fluorescence was identical in intact and broken cells expressing GFP or GFP fusions, allowing the use of culture sample fluorescence as an independent measure of expression. Results from stained band intensities were consistent with the far more precise fluorescence data.

**Figure 6:**
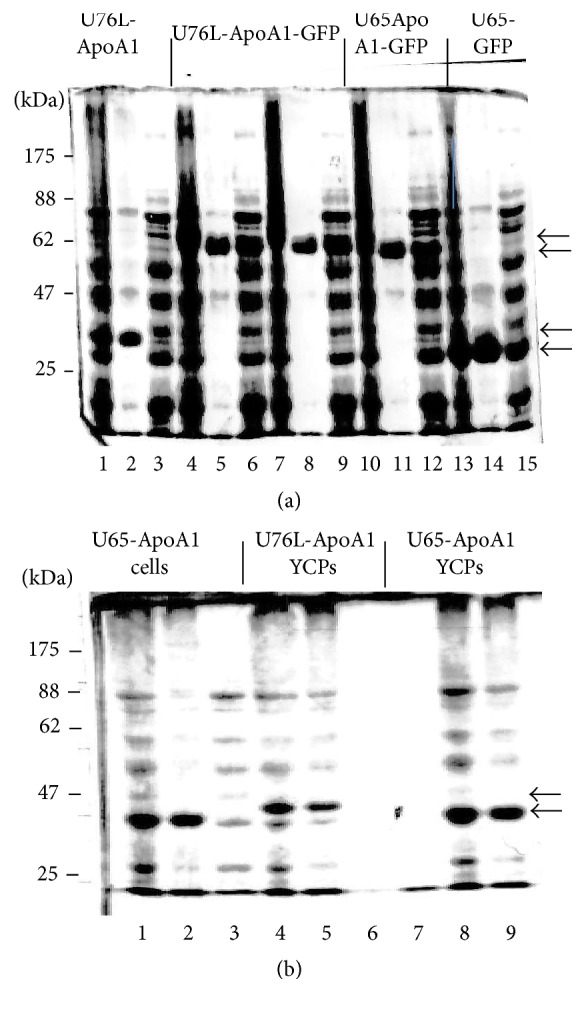
(a) SDS-PAGE of proteins from cells expressing the following: Lanes 1–3, U76L-ApoA1 (37.7 kDa, arrow); Lanes 4–6 and 7–9, U76L-ApoA1-GFP (64.6 kDa, arrow); Lanes 10–12, U65-ApoA1-GFP (62.5 kDa, arrow); and Lanes 13–15, U65-GFP (34.1 kDa, arrow). For each set, the first lane is 3,000 ×g supernatant, the second is 16,000 ×g pellet, and the third is 16,000 ×g supernatant. (b) SDS-PAGE. Lanes 1, 4, and 8 are total proteins, Lanes 2, 5, and 9 are 200,000 ×g pellets, and Lanes 3, 6, and 7 are 200,000 ×g supernatants. Lanes 1–3 and 7–9, cells and YCPs, respectively, expressing U65-ApoA1 (35.6 kDa, arrow). Lanes 4–6, YCPs expressing U76L-ApoA1 (37.7 kDa, arrow).

**Figure 7:**
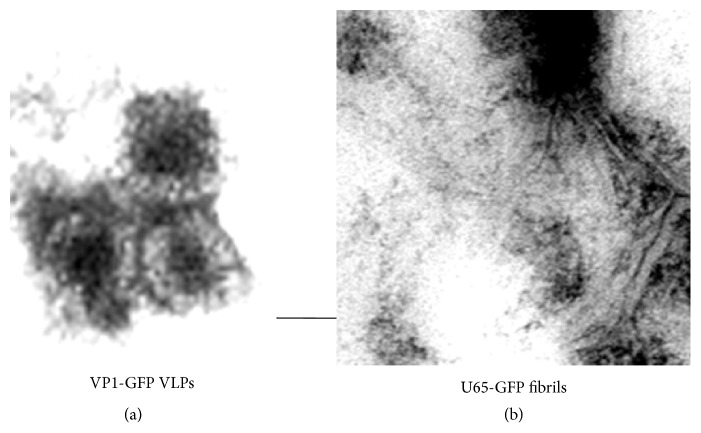
Negative-stain electron microscopy of (a) VP1-GFP VLPs and (b) aggregated fibrils of U65-GFP. The bar indicates 50 nm.

The U65 and U76L fusions formed single large aggregates in cells, visible by fluorescent microscopy (Figure 5(b)) and these were highly enriched in 16,000 ×g pellet fractions (Figure 6(a), Lanes 2, 5, 8, 11, and 14) derived from 3,000 ×g supernatants (Lanes 1, 4, 7, 10, and 13) of broken cells. Most yeast proteins remain in the 16,000 ×g supernatants (Lanes 3, 6, 9, 12, and 15). Cultures of independent U76L-ApoA1-GFP transformants (Lanes 4–9) illustrate the reproducibility of the expression data. After breaking cells in the presence of 40 mM octyl glucoside to solubilize membrane proteins and repeated rounds of differential centrifugation, SDS-PAGE analysis showed the GFP and ApoA1 fusion proteins with no visible contamination by yeast proteins (not shown). Electron microscopy of these purified fractions showed the linear fibrillar matrix characteristic of Ure2p aggregates (Figure 7(b)).

### 3.2. U65 and U76L Fusions Form an Aggregate with Yeast Proteins in pH 11.9 YCPs

The simplest and most scalable procedure for converting yeast cells to YCP vaccines is by extraction of mannoproteins for 12 min at 45°C at pH 11.9–11.7, a milder form of the process used for GP production [1]. Cells were rapidly killed and sterilized by this treatment.

After breakage of cells expressing U65-ApoA1 and pelleting wall fragments at 3000 ×g, total proteins are shown in Figure 6(b), lane 1. All of the U65-ApoA1 was found in the 200,000 ×g pellet from this fraction (lane 2) while most of the soluble yeast proteins remained in the 200,000 ×g supernatant (lane 3). Total proteins in broken U76L-ApoA1 and U65-ApoA1 YCPs processed under standard conditions are shown in lanes 4 and 8, respectively. All proteins were found in the 200,000 ×g pellet fraction (lanes 5 and 9). Little if any remained in the 200,000 ×g supernatant fractions (lanes 6 and 7). U65-GFP, U76L-GFP, and U65-ApoA1-GFP fusions fractionated similarly after pH 11.9 YCP processing (not shown). Thus U65 and U76L fusions form an insoluble aggregate with internal yeast proteins in pH 11.9-treated YCPs.

Removal of yeast cell membranes should allow elution of any soluble proteins through the exposed porous glucan shell. U65 and U76L fusions were retained in YCPs at 37°C in PBS in the presence of 1% triton X100 and 1% *β*-mercaptoethanol. Addition of 0.1% sarkosyl solubilized small quantities of low molecular weight yeast proteins. At higher sarkosyl levels, all proteins became progressively more soluble (not shown). Thus, yeast protein aggregates in YCPs could not be solubilized by nonionic detergents and the zwitterionic detergent disrupted both scaffolded antigens and protein aggregates. Denaturants (urea, guanidine hydrochloride), like detergents, failed to selectively solubilize host proteins.

### 3.3. Optimizing Glucan Exposure during YCP Preparation

**Figure 8:**
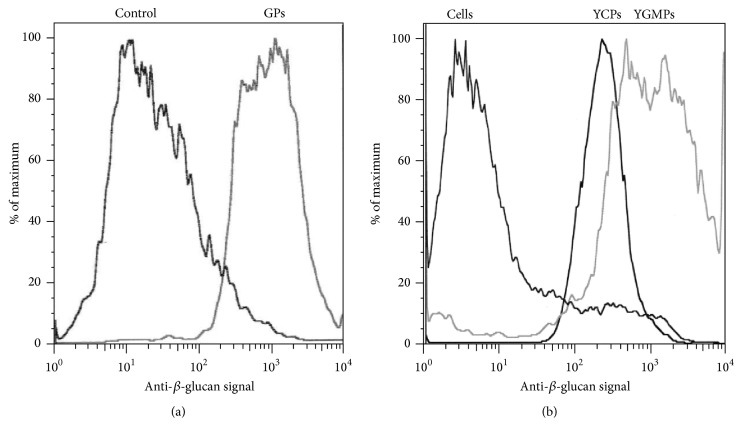
Anti-*β*-1,3-D-glucan binding (FACS). (a) Binding to* GPs* defines MFI (mean fluorescence intensity) of pure glucan.* Control*, binding to GPs using secondary antibody only. (b)* Cells*, untreated PAP1502 cells.* YCPs*, binding to pH 11.9 VP1-GFP YCPs.* YGMPs*, cell wall particles from which most mannan has been removed.

Glucan exposure on cells and YCPs was measured by binding of anti-*β*G monoclonal antibody using FACS analysis. As shown in Figure 8(a), purified GPs with fully exposed glucan had a mean fluorescence intensity (MFI) of ~1,500. Binding to YGMPs, heterogeneous glucan particles retaining a fraction of mannan [26], produced a broader fluorescence peak extending from about 400 to 1,500, equivalent to 25–100% of GP MFI (Figure 8(b)). Binding to untreated PAP1502 cells was very low (MFI 3-4), less than control GP samples stained with secondary antibody only (MFI 10, Figure 8(a)). YCPs exhibited sharp anti-*β*G binding peaks, indicating uniformity of mannan removal, as shown in Figure 8(b) for YCP-VP1-GFP (see below) made under standard pH 11.9 conditions. MFI for YCPs made at pH 11.9 varied between 12 and 15% of binding to GPs.

**Figure 9:**
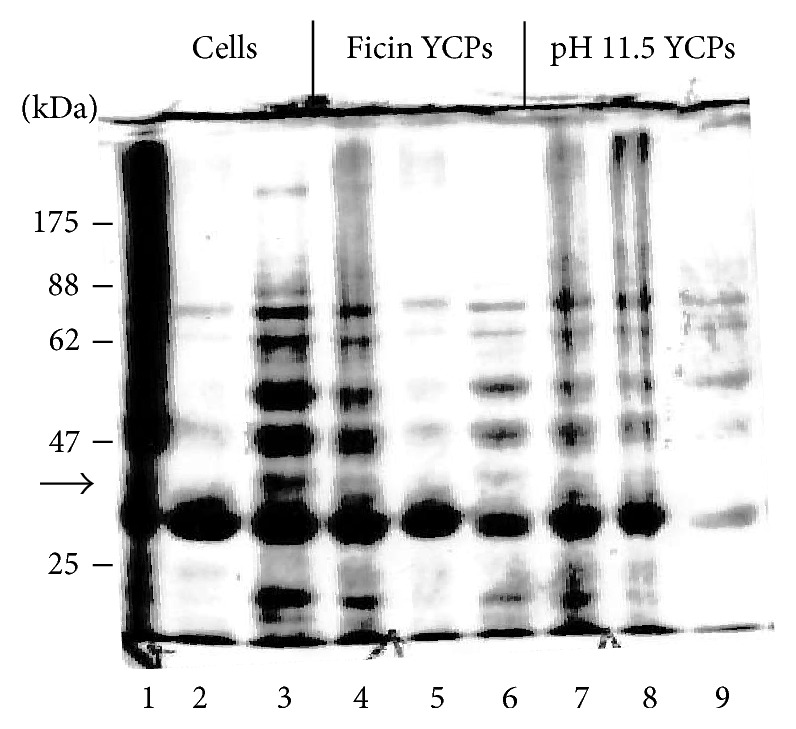
SDS-PAGE of proteins from cells and YCPs expressing U65-GFP (34.1 kDa, arrow). For each set, the first lane shows total proteins, the second is 16,000 ×g pellet, and the third is 16,000 ×g supernatant. Lanes 1–3, cells. Lanes 4–6, YCPs made by ficin hydrolysis. Lanes 7–9, YCPs made by hydrolysis at 45°C, pH 11.5 to 11.3.

U65-GFP YCPs were made by 12 min hydrolysis at 45°C using a range of initial pH values, hoping to find conditions compatible with both glucan exposure and retention of protein solubility. Binding to YCPs extracted at pH 12 to 11.8 was 20% of binding to GPs. At pH 11.9 to 11.7, binding was 14%. At pH 11.6 to 11.4, binding was 10%, and, at pH 11.5 to 11.3, binding was 6-7%. Thus, reduction in the stringency of alkaline hydrolysis produced a corresponding decrease in glucan exposure. However, even processing at pH 11.5 to 11.3 converted the majority of yeast proteins and the antigen into a common aggregate present in the 16,000 ×g pellet (Figure 9, Lane 8), although about 30% did remain soluble (Lane 9).

### 3.4. GFP Is Effectively Trapped by U65 and U76L Scaffolds at pH 11.5

While fluorescence of GFPbex1 is remarkably stable at pH 7 to 11.4 at 23°C, fluorescence is completely lost during YCP processing at 45°C at an initial pH of 11.7 or higher. Processing of cells at an initial pH of 11.5, however, producing 6-7% glucan exposure, allowed 30–50% preservation of fluorescence in cells expressing free GFP, U65-GFP, or U76L-GFP. U65-GFP remains visible as a single aggregate (Figure 5(c)), much like that present before YCP processing (Figure 5(b)). 8% of free GFP and 2–4% of U65-GFP or U76L-GFP fluorescence were released during processing. SDS-PAGE analysis of the proteins released showed only free GFP, presumably cleaved from the scaffolded GFP during the 18–20-hour period of galactose-induced expression. While a subsequent wash with 1% Triton X100 in PBS + 1%  *β*-mercaptoethanol released almost all of the residual free GFP, no additional U65-GFP or U76L-GFP fluorescence was released. The U65 and U76L scaffolds, therefore, effectively retained linked GFP during YCP processing at pH 11.5.

### 3.5. Loss of Surface Mannan in YCPs and Glucan Exposure Revealed by 3T3-D1 Cell Uptake

Phagocytosis of YCPs by 3T3 cells expressing the dectin-1 *β*-1,3-glucan receptor (3T3-D1 cells), surrogate APCs, is a much less sensitive but functionally more relevant test of glucan exposure than anti-glucan antibody binding. Against a high 25% background for untreated cells, uptake of YGMP (positive controls for high glucan exposure) and YCPs with 14% glucan exposure was 90–95%, and uptake of YCPs with 10% glucan exposure was about 90%. Uptake was reduced to 80–85% in YCPs processed at pH 11.5 with 6-7% glucan exposure and to 70–75% in ficin-treated YCPs (see below) with 5-6% exposure. Mannan loss under standard YCP processing conditions was assessed by staining with Alexa Fluor 488-labeled Concanavalin A, a lectin that selectively binds to mannan. Binding was reduced by 38% after 10 min and 45% after 15 min, indicating loss of a large fraction of surface-accessible mannoprotein.

### 3.6. Use of Protease for Mannoprotein Removal and YCP Production

Hydrolysis of PAP1502 cells for 30 min at 37°C with 2, 4, or 8 mg/mL ficin resulted in increasing glucan exposure. A wash at 23°C with 20 mM NaOH at pH ~12 then killed and sterilized the cells and exposed additional glucan, but membranes apparently remained intact since GFP fluorescence was unchanged and internal yeast proteins remained in the 16,000 ×g supernatant after breakage (Figure 9, Lane 6). U65-GFP remained in an aggregate, mostly found in the 16,000 ×g pellet (Lane 5). Maximal glucan exposure, after 8 mg/mL ficin hydrolysis, was 5-6% of GP levels. Only yeast proteins less than about 30 kDa were eluted by washes of ficin-treated YCPs with PBS + 1% Triton X100, 0.1% sarkosyl, and 1%  *β*-mercaptoethanol (not shown). Thus, the porosity of the exposed glucan remained severely compromised by residual mannan and a single ficin treatment failed to render cell walls sufficiently permeable for effective removal of yeast proteins.

### 3.7. Mouse Responses to a U76L-ApoA1 YCP Vaccine

**Figure 10:**
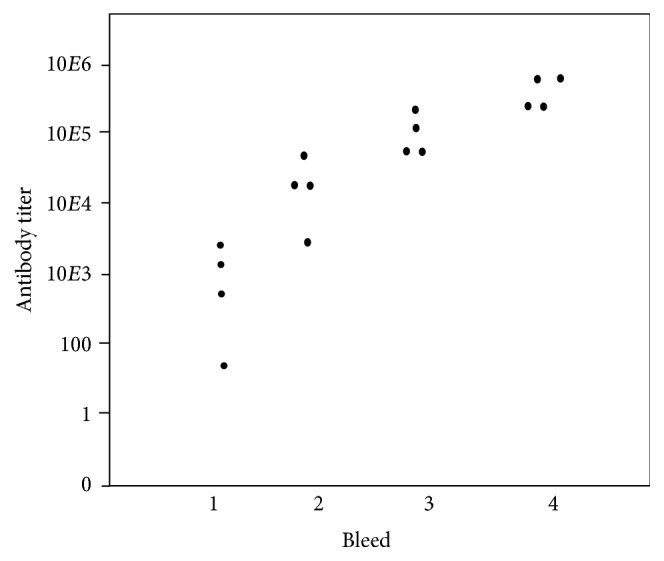
Anti-ApoA1 IgG ELISA titers after subcutaneous YCP-U76L-ApoA1 vaccination. YCPs containing about 90 *μ*g ApoA1 were administered to four mice on days 0, 14, 28, and 42. Bleeds were on days 13, 27, 41, and 55.

Four C57BL/6 mice were vaccinated subcutaneously with 2 × 10^8^ U76L-ApoA1 YCPs in which ApoA1 represented about 8% of total protein (Figure 6(a)), and glucan exposure was about 14% that of GPs. Doses contained about 90 *μ*g ApoA1 and 1.2 mg yeast proteins. Bleeds were made after 13 days and subsequent doses were administered a day later. IgG responses to ApoA1 determined by ELISA (Figure 10) increased nearly exponentially for three doses and averaged 400,000 reciprocal titer after the fourth dose. IgG responses to total yeast proteins increased in parallel and were about 20% of ApoA1 responses (not shown). ELISA responses using wells coated with 0.1 *μ*g U76L-GFP, reflecting response to the U76L scaffold, were less than 1% of responses to ApoA1 (not shown).

### 3.8. Dependence of Mouse Responses to U65-GFP YCPs on Glucan Exposure and Dose

**Figure 11:**
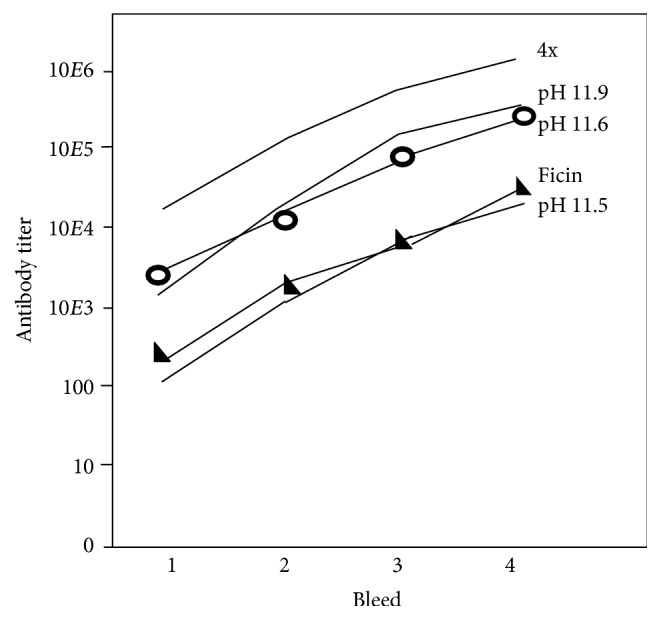
Average anti-GFP IgG ELISA titers after subcutaneous YCP-U65-GFP vaccination. Grey triangles: mice vaccinated with YCPs from ficin-treated cells. YCPs from cells processed at pH 11.5 gave very similar data. Open circles: mice vaccinated with YCPs from cells processed at pH 11.6. Mice vaccinated with YCPs from cells processed at pH 11.9 gave very similar data. The upper line, mice vaccinated with 4x doses of pH 11.9 YCPs.

Cells expressing the U65-GFP fusion protein at 8% of total protein were converted to YCPs by extraction at 45°C at either pH of 11.9–11.6, 11.6–11.4, or 11.5 to 11.3, or by hydrolysis with 8 mg/mL ficin. Glucan exposures were 15, 10, 7, and 6% of GPs, respectively. The pH 11.9 vaccine was tested at two dose levels, 1x and 4x the dose used for all other vaccine formulations. Groups of five mice were vaccinated subcutaneously at two-week intervals. Doses contained about 1.2 mg total protein and 90 *μ*g GFP, except for the 4x group. Average GFP-specific IgG titers are shown in Figure 11. Variance between titers for the four mice at each point was no more than 16 fold. A 4x dose of pH 11.9 YCPs elicited the strongest average responses (upper line). Bleed 4 titers were uniformly at least 10^6^. A 1x dose produced a 5-fold weaker response. YCPs extracted at pH 11.6 with 10% glucan exposure were of nearly comparable efficacy (open circles). YCPs extracted at pH 11.5 or by treatment with ficin (triangles), with 6-7% glucan exposure, were about 10-fold less effective than those processed at pH 11.9 or 11.6.

### 3.9. Expression of MPyV VP1 and VP1-GFP Fusions

**Figure 12:**
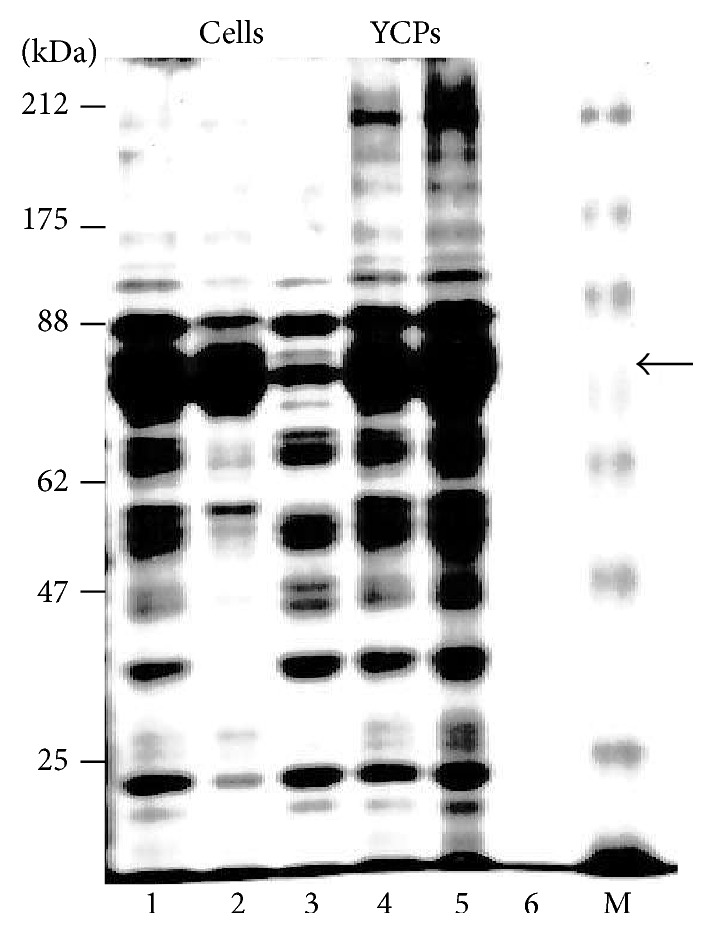
SDS-PAGE of proteins from cells (Lanes 1–3) and YCPs (Lanes 4–6) expressing VP1-GFP (70 kDa, arrow, 12% of total protein). Lanes 1 and 4, total proteins. Lanes 2 and 5, 16,000 ×g pellet. Lanes 3 and 6, 16,000 ×g supernatant. M: marker proteins.

MPyV strain BG (previously identified as strain A2) has been extensively used for analysis of immune responses in mice [27]. The VP1 major capsid protein gene was cloned in pB4 by PCR using a virus stock as template. Following expression in strain CRY1, Western blots using polyclonal mouse serum raised against MPyV showed the predicted 42 kDa monomer VP1 and 200 kDa pentamer VP1 bands. These bands were completely retained during YCP processing for up to 16 min at 45°C, at pH 11.9 to 11.7 (not shown). GFP was fused independently to the N- or C-terminus of VP1. Optimal expression levels of both were 30% of total protein, as shown for the 70 kDa VP1-GFP band in Figure 5(a), Lane 1, and Figure 12, Lane 1. As would be expected for VLPs, most of the VP1-GFP was in the 16,000 ×g, 25 min pellet from the 3,000 ×g supernatant (Figure 12, Lane 2), rather than in the supernatant (Lane 3). Following YCP processing, all VP1-GFP and yeast proteins remained in the YCPs (Lane 4) and, after breakage, all proteins were retained in the 16,000 ×g pellet (Lane 5), apparently as a common aggregate. A very little amount remained soluble (Lane 6). VP1-GFP was enriched from cells broken in the presence of octyl glucoside; after two rounds of differential centrifugation, SDS-PAGE analysis showed purity exceeding 95% (not shown). Negative-stained electron microscopy showed abundant and uniform 80–90 nm diameter particles (Figure 7(a)). VP1 monomers are 50 kDa and their formation involves interaction of N- and C-termini [17], presumably precluded in the GFP fusions. These particles appear to be discrete VLP-like VP1 aggregates, probably with surface GFP decoration.

### 3.10. Antibody Responses to Oral and Subcutaneous Vaccination with YCP-VP1-GFP

YCPs made from cells expressing VP1-GFP at 12% of total protein were administered orally or subcutaneously to compare the efficacy of these two common routes of vaccination in protection against MPyV infection. Group A mice were vaccinated subcutaneously with YCPs containing 96 *μ*g VPI-GFP fusion protein and received four boosts at 14-day intervals. As described for oral starch microparticle vaccines [24], Groups B and C mice were vaccinated orally on three consecutive days with YCPs containing, respectively, about 400 and 960 *μ*g VPI-GFP fusion protein, a total of 12.5 and 30 times the subcutaneous dose. Group D mice were unvaccinated controls. Bleeds for analysis of antibody responses were made on the day prior to each vaccine boost and 14 days after the final doses. Stool was collected for analysis of IgA production on day 56 before final boost 4. Finally, to test* in vivo* protection against MPyV infection, mice were infected intraperitoneally with 10^6^ pfu/mouse on day 71.

**Figure 13:**
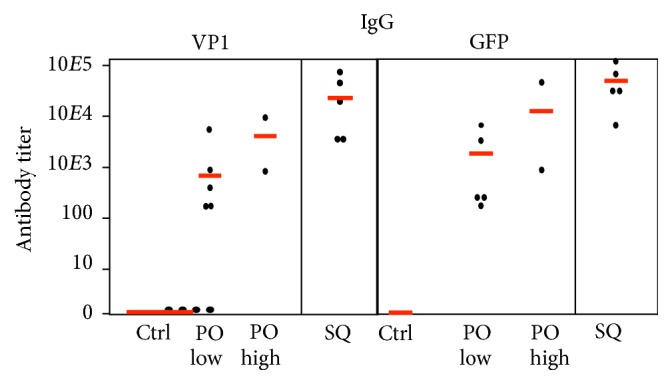
VP1-specific (left) and GFP-specific (right) IgG titers (ELISA) in sera from bleed 5 in mice vaccinated with VP1-GFP YCPs. Vaccine was administered orally (PO) at lower (3x 400 *μ*g VP1-GFP) and higher (3x 960 *μ*g VP1-GFP) doses or subcutaneously (SQ) at a 96 *μ*g dose. Control (Ctrl) mice were not vaccinated. Red lines indicate mean values.

Anti-VP1 IgG responses were easily detectable on day 28 and the IgG titers approximately doubled after each dose (Table 1), averaging 12,000 on day 71 (Figure 13). Reponses to oral doses were easily detectable on day 42 and also approximately doubled after each later dose (Table 1). The lower dose produced titers about 10% of those produced by subcutaneous vaccination. The high dose responses were about twofold higher, averaging 20% of those elicited by subcutaneous dosage (Figure 13). Three mice in the high dose oral group died shortly after administration of the initial dose, most likely caused by trauma associated with the high volume gavage, leaving only two. IgG responses to GFP following subcutaneous dosage were first detectable at day 14 and were about twice those to VP1 at all later time points, showing a similar doubling with subsequent boosts to an average titer of 25,000 on day 71 (Table 2, Figure 13). GFP-specific responses to oral doses were detectable on day 28. Responses to oral doses were two- to fourfold higher than responses to VP1, as shown for day 71 data (Table 2, Figure 13). IgG2B responses to VP1 and GFP were very similar (not shown).

The sum total of IgG responses to the many yeast proteins that constituted 88% of the total proteins in the YCP-VP1-GFP preparation was about twice those to VP1 and similar to anti-GFP responses. Western blots using sera from bleed 5 of mice immunized subcutaneously with VP1-GFP YCPs, against total proteins from strain PAP1502 yeast cells expressing VP1-GFP, showed responses to multiple unidentified yeast proteins, predominantly species ~60 kDa and higher (not shown). The most prominent band apparently corresponded to VP1-GFP (70 kDa). Responses to mannoproteins, which migrate as unresolved species >100 kDa, were relatively weak.

**Figure 14:**
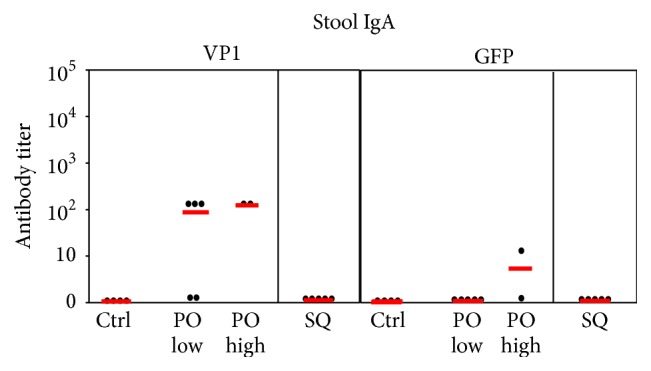
IgA responses to VP1 and GFP in stools at day 56. Mouse groups are as described in Figure 13. Red lines indicate mean values.

Anti-GFP IgA in stool on day 56 was undetectable after subcutaneous or lower dose oral vaccination and only detectable at dilution >8 in one mouse after higher dose oral vaccination (Figure 14). Anti-VP1 IgA was undetectable after subcutaneous vaccination, but the response to oral vaccination was >100 at either dose level in 5 of 7 mice (Figure 14). Thus, oral YCP-GFP-VP1 vaccination elicited mucosal IgA antibody responses preferentially to VP1.

### 3.11. The YCP-VP1 Vaccine Protects Mice against MPyV Infection

While MPyV infection in immunodeficient mice can be lethal, in immunocompetent mice, infection follows a predictable, self-limiting course [27, 28]. Virus replicates rapidly in multiple organs, resulting in peak viral loads on days 5-6 after infection. After this time, an adaptive immune response initiates viral clearance leading to reduction of the virus load, but lifelong low levels of PyV persistence remain. In mice immunized with YCP-VP1-GFP, antiviral serum antibodies and VP1-specific memory T and B cells are predicted to reduce the peak virus load following challenge with live MPyV. Protection against MPyV infection in these mice was assessed by measuring viral DNA genome copies/*μ*g organ DNA 5 days after MPyV infection, in comparison with MPyV-infected, unimmunized mice. qPCR measurements of MPyV genome copies correlate with measurements of infectious virus load in organs by plaque assays [29].

**Figure 15:**
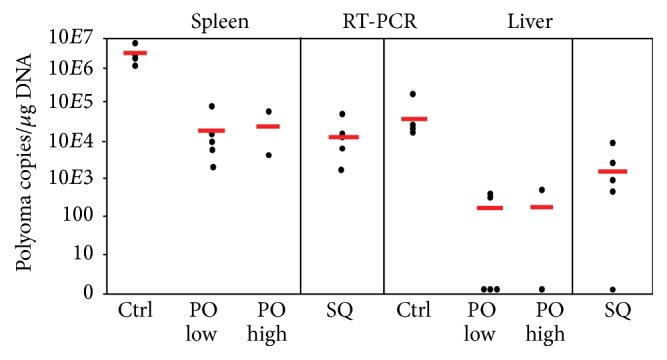
Viral load expressed as MPyV DNA genome levels in organs of YCP-VP1-GFP immunized and unimmunized (Ctrl) mice 5 days after MPyV infection. Mouse groups are as described in Figure 13. Red lines indicate the mean values.

Mice were infected intraperitoneally 15 days after the fifth vaccine dosage, one day after bleed 5, and were sacrificed 5 days later. Viral load (Figure 15) was assayed by measuring viral genome copies in spleen and liver tissues. In spleen, viral genome copies/*μ*g DNA in the unvaccinated controls ranged from 2.6 × 10^6^ to 9.3 × 10^6^ (mean 5.3 × 10^6^). This was reduced in subcutaneously vaccinated mice to between 3,600 and 101,000 (mean 28,600) and in orally vaccinated mice to 8,000 to 160,000 (lower dose, mean 73,000) and 23,000 to 91,000 (higher dose, mean 57,000). Reductions in spleen viral load after both subcutaneous and oral immunization were statistically significant (*p* = 0.03 and *p* = 0.035, resp.). The viral load in liver tissues was 200-fold lower than in spleen, 12,600 to 31,900 in the control unvaccinated mice (mean 24,100) and <20 to 3,700 in the subcutaneously vaccinated mice (mean 970). In the orally vaccinated mice, half of which had no detectable viral transcripts, viral load at low dose was <20 to 570 (mean 178) and at the high dose was <20 to 820 (mean 410). Thus, both subcutaneous and oral vaccination with VP1-GFP YCPs reduced spleen and liver viral replication levels by at least two logs.

## 4. Discussion

Glucan particle (GP) (3) and Yeast Cell Particle (YCP) vaccines provide novel vaccine antigen-adjuvant delivery systems with the potential for improved vaccine efficacy and reduced antigen dose. Processing of antigen-containing yeast cells into YCP vaccines requires removal of surface mannoproteins, exposing the *β*-1,3-glucan to promote efficient glucan receptor-mediated phagocytosis by APCs [4]. Most of the abundant and dominant yeast cell wall epitopes [10] should be concomitantly removed. As the glucan shell is highly permeable, we anticipated that retention of soluble antigens during YCP processing would require anchoring to cytoplasmic aggregates. This was demonstrated for residual fluorescent, scaffold-free GFP in YCPs processed at pH 11.5, and is likely to be essential for YCPs processed by the standard pH 11.9 procedure if the antigens are small proteins or peptide fragments. However, a scaffold is probably unnecessary at pH 11.9 for larger antigens, since such proteins are converted to a denatured aggregate. If YCP processing by ficin treatment, where native protein folds are preserved, can be improved to achieve the >10% glucan exposure required for optimal APC interaction, this would potentially allow nonionic detergent elution of soluble yeast proteins and a scaffold would be essential for antigen retention.

Virus-like particles formed in yeast by self-assembly of MPyV VP1 viral capsids [17] are effective GFP scaffolds. IgG responses to the VP1 component of the VP1-GFP vaccine were about half of those to GFP. The much smaller 7.2 kDa U65 self-assembling peptide was predicted to be compatible with any linked antigen and to induce much weaker competing antiscaffold immune responses. U76L (9.3 kDa), with 11 additional Ure2p residues and a flexible linker L, was designed as a potentially more stable scaffold, presumably permissive of independent folding of any linked antigen. U65- and U76L-GFP and ApoA1 fusions were highly expressed and formed large aggregates found in 16,000 ×g pellets from broken cells and the GFP fusions were readily visualized in intact cells and pH 11.5 YCPs. MHCII binding predictions in C57BL/6 mice for both scaffolds are relatively weak [14, 15], and while responses to the U76L and U65 scaffolds were not independently tested, responses to U76L-GFP (which includes the U65 scaffold) in mice vaccinated with U76L-ApoA1 were less than 1% of responses to the ApoA1 antigen.

U76L-ApoA1 and U65-GFP pH 11.9 YCP vaccines were highly effective in mice by subcutaneous administration. IgG responses to four doses of U76L-ApoA1 YCPs approached 10^6^ reciprocal dilution titer. Responses to U65-GFP YCPs were similar, while antibody responses to the 12-fold excess of total yeast proteins were about 5-fold weaker. The dominant immune responses to the ApoA1 and GFP antigens presumably result from higher abundance and polymeric association with the peptide scaffolds. U65-GFP YCP vaccines with about 10% glucan exposure were as effective as those with 14 or 20% exposure; however, YCPs with only 6-7% exposure, whether produced by hydrolysis at lower pH or by ficin treatment, were 5–10-fold less effective. Glucan exposure below 10%, therefore, resulted in proportionately reduced vaccine efficacy, implying that its principal determinant is interaction with APC glucan receptors, as reflected in 3T3-D1 cell uptake efficiency. While U65 and U76L fusions behaved very similarly in mice, expression of U65-fusions was consistently 10 to 15% higher than equivalent U76L fusions, and no evidence for differences in folding or stability of U65- and U76L-GFP fusions was observed in cells, during YCP production or in subcellular fractions. The simpler U65 scaffold, therefore, appears to be superior.

Parenteral administration of the purified MPyV VP1 capsid protein elicited IgG antibodies in mice [27, 28] and provided partial protection upon MPyV challenge, reducing peak virus load [29]. Hamster PyV (HaPyV) VLPs are also known to be good immunogens and are used in a recombinant form to generate antibody responses to a variety of antigens [18]. Here, we test the ability of YCP-VP1-GFP oral vaccination in mice to protect against systemic MPyV infection. The utility of GPs in subcutaneous delivery of purified antigens is well established [3]. Parenteral vaccinations, however, often fail to generate the effective mucosal immune responses required to combat common intestinal (and other mucosal) infections, and development of an effective oral vaccination route would be a major advance, particularly in developing countries [30]. Oral vaccination may also elicit serum IgG responses, providing protection against a broad range of infections, and an oral viral capsid-expressing yeast vaccine has been shown to elicit neutralizing IgG in mice, superior to purified capsid antigen [31]. An orally administered YCP-VP1-GFP vaccine elicited VP1-specific intestinal IgA and serum IgG responses and we observed reduction in MPyV load in liver and spleen tissues by 98-99% after systemic (intraperitoneal) challenge of the vaccinated mice with live virus. Peak levels at day 5 after infection were well below the lifelong levels of viral persistence seen in immune-competent mice following infection [29]. While multiple oral doses were administered before protection was tested, this demonstration of effective protection against systemic viral infection is clearly promising, especially for livestock, where use of YCPs in multiple doses as a feed supplement is possible.

Selective removal of yeast proteins from YCPs would potentially prevent sensitization of vaccine recipients to dietary yeast [12]. Processing at high pH produces YCPs with denatured and aggregated internal proteins, preventing selective elution. In YCPs produced by ficin treatment, all internal proteins apparently remained in their native state, potentially allowing responses to topological epitopes and facilitating selective elution of yeast proteins. This, however, was not achieved after a single ficin treatment. More vigorous proteolysis conditions may increase glucan exposure to the 10% required for optimal vaccine function while allowing removal of yeast proteins soluble in nonionic detergents.

Several reports describe testing of whole recombinant antigen-expressing yeast cells, retaining all of their internal proteins and mannoproteins, as vaccines. Heat-killed yeast expressing carcinoembryonic antigen (CEA), administered to patients with metastatic, CEA-expressing carcinomas, was well tolerated [32]. In mouse models, yeast expressing HCV NS3-Core fusion protein induced antigen-specific cytotoxic T cell responses that resulted in eradication of circulating cells expressing the antigen [33, 34], and yeast expressing mutant BCR-ABL oncogene protein, used as a subcutaneous vaccine, eliminated leukemia cells expressing this mutant protein [35]. Recombinant yeast has also been used in oral vaccines intended for livestock use [8]. Most definitively, oral vaccination with heat-killed* K. lactis* or* P. pastoris* expressing infectious bursal disease virus VP2 capsid elicited protective responses in chickens, preventing mortality and reducing lesions [12, 36].

## 5. Conclusion

YCP vaccines expressing viral capsid VLPs, scaffolded antigens or, potentially, scaffolded peptide epitopes, can be produced rapidly, should be easily stored, and are highly effective in mice, eliciting strong IgG responses and providing protection against a polyoma virus challenge by either subcutaneous or oral dosage. While current formulations may not be suitable for human use, they have clear potential for use in feed animal vaccines.

## Figures and Tables

**Table 1:** Anti-VP1 IgG titers, defined as reciprocals of dilutions giving a response twofold above background.

Vaccine dosage	Bleed 1	Bleed 2	Bleed 3	Bleed 4	Bleed 5
(A) Subcutaneous	<200	400–800	800–6400	1600–6400	3200–51200
Average	<200	650	2,800	5,600	12,000
(B) Low dose oral	<200	<200	200–400	400–1600	200–3200
Average	<200	<200	320	800	960
(C) High dose oral	<200	<200	200–400	800–1600	800–3200
Average	<200	<200	350	1200	2000

**Table 2:** Anti-GFP IgG titers, defined as reciprocals of dilutions giving a response twofold above background.

Vaccine dosage	Bleed 1	Bleed 2	Bleed 3	Bleed 4	Bleed 5
(A) Subcutaneous	400–1600	800–6,400	3,200–12,8000	1,600–25,600	3,400–102,000
Average	800	3,000	6,000	10,000	25,000
(B) Low dose oral	<200	200–800	400–6400	400–3200	200–3200
Average	<200	400	2200	1200	1600
(C) High dose oral	<200	200–800	200–12,800	400–12,800	400–12,800
Average	<200	500	3,500	6,700	6,700
